# Loss of interneurons and disruption of perineuronal nets in the cerebral cortex following hypoxia-ischaemia in near-term fetal sheep

**DOI:** 10.1038/s41598-018-36083-y

**Published:** 2018-12-06

**Authors:** Tania M. Fowke, Robert Galinsky, Joanne O. Davidson, Guido Wassink, Rashika N. Karunasinghe, Jaya D. Prasad, Laura Bennet, Alistair J. Gunn, Justin M. Dean

**Affiliations:** 1 Department of Physiology, Faculty of Medical and Health Sciences, University of Auckland, Auckland, New Zealand; 2 The Ritchie Centre, Hudson Institute of Medical Research, Melbourne, Australia

## Abstract

Hypoxia-ischaemia (HI) in term infants is a common cause of brain injury and neurodevelopmental impairment. Development of gamma-aminobutyric acid (GABA)ergic circuitry in the cerebral cortex is a critical event in perinatal brain development. Perineuronal nets (PNNs) are specialised extracellular matrix structures that surround GABAergic interneurons, and are important for their function. Herein, we hypothesised that HI would reduce survival of cortical interneurons and disrupt PNNs in a near-term fetal sheep model of global cerebral ischaemia. Fetal sheep (0.85 gestation) received sham occlusion (n = 5) or 30 min of reversible cerebral ischaemia (HI group; n = 5), and were recovered for 7 days. Expression of interneurons (glutamate decarboxylase [GAD]^+^; parvalbumin [PV]^+^) and PNNs (*Wisteria floribunda* agglutinin, WFA) was assessed in the parasagittal cortex by immunohistochemistry. HI was associated with marked loss of both GAD^+^ and PV^+^ cortical interneurons (all layers of the parasagittal cortex and layer 6) and PNNs (layer 6). The expression and integrity of PNNs was also reduced on surviving GAD^+^ interneurons. There was a trend towards a linear correlation of the proportion of GAD^+^ neurons that were WFA^+^ with seizure burden (r^2^ = 0.76, *p* = 0.0534). Overall, these data indicate that HI may cause deficits in the cortical GABAergic system involving loss of interneurons and disruption of PNNs, which may contribute to the range of adverse neurological outcomes following perinatal brain injury.

## Introduction

Perinatal hypoxic-ischemia (HI) is a common trigger of brain injury in term infants, being responsible for approximately 1–3 cases of moderate to severe encephalopathy per 1,000 live births, and is associated with a high risk of death or disability^1^. A major neuropathology observed following HI at term involves parasagittal watershed zone injury with neuronal loss, and damage to the underlying subcortical white matter^2–9^. This pattern of injury is strongly associated with adverse neurological outcomes, including cerebral palsy, cognitive delay, and epilepsy^10–14^.

During late prenatal and early postnatal development, the brain undergoes a period of marked growth and wiring, resulting in the establishment of highly ordered and complex functional networks^15^. A major aspect of this circuit development involves the integration of gamma-aminobutyric acid (GABA)ergic interneurons into the cerebral cortex^16,17^. GABAergic interneurons are the major population of cortical inhibitory neurons^18^, representing approximately 20% of all cortical neurons^19^. GABAergic circuits provide a critical source of inhibition required for the regulation of neuronal signalling, and thus maintain the balance between excitatory and inhibitory activity^20,21^. Importantly, a major period of GABAergic network development in the cerebral cortex occurs during the late prenatal period^22,23^. Thus, injury to the brain during this time has the potential to affect GABAergic circuitry and cortical function. Indeed, there is limited evidence for loss and dysfunction of interneurons in the cerebral cortex in human post-mortem brain tissue following perinatal brain injury^24^ and in experimental rodent models of neonatal HI^25–27^.

Perineuronal nets (PNNs) are specialised structures, formed by dense mesh-like aggregates of multiple extracellular matrix (ECM) molecules, which enwrap interneurons in the cerebral cortex and other brain regions^28,29^. PNNs are commonly detected using lectins such as *Wisteria floribunda* agglutinin (WFA) that bind to the N-acetylgalactosamine residues of chondroitin sulphate proteoglycans^30,31^. PNNs are important for normal GABAergic neuron function^32–34^, including the regulation of cortical synaptic formation and stabilisation^35–37^, and have key roles in neuronal plasticity throughout development (reviewed in^38^). Alterations in PNN formation and integrity are also associated with various human neurological diseases including schizophrenia^39,40^, epilepsy^41,42^, autism and Rett Syndrome^43^; PNN disruption was proposed to contribute to the associated deficits in neuronal signalling and cognition/behaviour in these disorders. Further, loss of cortical PNNs was reported in adult sheep and rodents following focal ischemia^44,45^.

The effects of perinatal HI on cortical interneurons and PNNs remain unclear. Thus, in the present study we tested the hypothesis that cortical PNNs are associated with interneurons in the near-term fetal sheep, and that global cerebral ischaemia is associated with loss of both cortical interneurons and PNNs.

## Methods

### Animal experiments

#### Fetal surgery

All procedures were approved by the Animal Ethics Committee of the University of Auckland under the New Zealand Animal Welfare Act, and the Code of Ethical Conduct for animals in research established by the Ministry of Primary Industries, Government of New Zealand. Fetal surgery and instrumentation were previously described^46^. In brief, time-mated Romney/Suffolk fetal sheep were instrumented using sterile techniques at 118–124 h gestation (term is 145 d. Sheep at this age (0.85 gestation) are comparable to the term human infant in terms of brain maturation^47,48^. Food, but not water was withdrawn 18 h before surgery. Ewes were given long acting oxytetracycline (20 mg/kg intramuscular; Phoenix Pharm, Auckland, New Zealand) at 30 min before the start of surgery. Anaesthesia was induced by injection of propofol (5 mg/kg intravenous [i.v.]; AstraZeneca Limited, Auckland, New Zealand) and maintained using 2–3% isoflurane in oxygen. The depth of anaesthesia, maternal heart rate, and respiration were constantly monitored by trained anaesthetic staff. Ewes received a constant infusion isotonic saline drip (infusion rate approximately 250 mL/h) to maintain fluid balance.

Following a maternal midline abdominal incision, the fetus was exposed and both fetal brachial arteries were catheterised with polyvinyl catheters to measure mean arterial blood pressure. An amniotic catheter was secured to the fetal shoulder. Electrocardiographic electrodes (Cooner Wire Co., Chatsworth, CA, USA) were sewn across the fetal chest to record fetal heart rate. The vertebral-occipital anastomoses were ligated, and inflatable carotid occluder cuffs were placed around both carotid arteries^49,50^. A 3S Transonic ultrasonic flow probe (Transonic systems, Ithaca, NY, USA) was placed around the right carotid artery. Using a seven-stranded stainless steel wire (AS633–7S’s SF; Cooner Wire Co.), two pairs of electroencephalographic (EEG) electrodes were placed on the dura over the parasagittal parietal cortex (10 mm and 20 mm anterior to bregma, 10 mm lateral) and secured with cyanoacrylate glue. A reference electrode was sewn over the occiput. A further two electrodes were sewn in the nuchal muscle to record electromyographic activity as a measure of fetal movement. The uterus was then closed and antibiotics (80 mg gentamicin; Pharmacia and Upjohn, Rydalmere, NSW, Australia) were administered into the amniotic sac. The maternal laparotomy skin incision was infiltrated with 10 mL 0.5% bupivacaine plus adrenaline (AstraZeneca Ltd., Auckland, New Zealand). All fetal catheters and leads were exteriorised through the maternal flank. The maternal long saphenous vein was catheterised to provide access for post-operative maternal care and euthanasia.

#### Post-operative care

Sheep were housed together in separate metabolic cages with access to food and water *ad libitum*, and kept in a temperature-controlled room (16 ± 1 °C, humidity 50 ± 10%) with a 12 h light/dark cycle. Antibiotics were administered daily for 4 d to the ewe (600 mg benzylpenicillin sodium i.v.; Novartis Ltd., Auckland, New Zealand; and 80 mg gentamicin i.v.). Fetal catheters were maintained patent by continuous infusion of heparinised saline (20 U/mL at 0.15 mL/h), and the maternal catheter maintained by daily flushing.

#### Data recording and blood sampling

Data recordings began 24 h prior to the start of the experiment and continued for the remainder of the experiment. The analogue fetal EEG signal was low pass filtered with a cut-off frequency set with the −3 dB point at 30 Hz, and digitised at a sampling rate of 512 Hz. EEG power was derived from the power spectrum signal between 0.5 and 20 Hz^51^, and was normalised by log transformation (dB, 20 × log power). Data were recorded and saved continuously to disk for off-line analysis using custom data acquisition programs (LabView for Windows; National Instruments, Austin, TX, USA). Arterial blood samples were taken for pre-ductal pH, blood gas, base excess (Ciba-Corning Diagnostics 845 blood gas analyser and co-oximeter; Medfield, MA, USA), glucose, and lactate measurements (YSI model 2300; Yellow Springs, OH, USA). All fetuses had normal biochemical variables for their gestational ages^52,53^.

#### Experimental protocols

Fetuses were randomised to cerebral ischaemia (HI group; n = 5) or sham occlusion (control group; n = 5). At 128 ± 1 d gestation, ischaemia was induced by reversible inflation of the carotid occluder cuffs with sterile saline for 30 min. Successful occlusion was confirmed by the onset of an isoelectric EEG signal within 30 s of inflation. The carotid occluder cuffs were not inflated in sham control experiments. Fetal blood samples were drawn just before the occlusion, and at 2 h, 4 h, and 6 h after occlusion, followed by daily sampling for the remainder of the experiment. Animals were euthanized at 7 d recovery with an overdose of sodium pentobarbitone (9 g i.v. to ewe; Pentobarb 300; Chemstock International, Christchurch, New Zealand).

### Immunohistochemistry

All antibodies/markers used in this study are summarised in Table 1.

**Table 1:** Antibodies and markers used for immunolabelling studies.

Antibody	Concentration	Specificity	Supplier details
Calmodulin-dependent protein kinase IIα (CaMKIIα)	1:200	Excitatory neurons	#C6974; Sigma-Aldrich
CTIP2	1:500	Layer 5 cortical neurons; weaker expression in layer 6 neurons	#ab18465; Abcam
Gamma-aminobutyric acid (GAD)65/67	1:200	GABAergic interneurons	#ab49832; Abcam
Parvalbumin (PV)	1:50	PV interneuron subpopulation	#ab11427; Abcam
Myelin basic protein (MBP)	1:500	Myelin	#MBP; Aves Labs
NeuN	1:20	All post-mitotic neurons	#MAB377; Merck Millipore
Biotinylated *Wisteria floribunda* agglutinin (WFA)	1:400	Perineuronal nets	#L1516; Sigma-Aldrich

#### Diaminobenzidine labelling of glutamate decarboxylase

At post-mortem, fetal sheep brains were perfusion fixed *in situ* in 10% phosphate-buffered formalin. Brains were then embedded in paraffin, cut into 10 µm thick sections using a microtome (Leica Jung RM2035; Leica Microsystems, Albany, New Zealand), and mounted onto glass slides. Regions of the forebrain used for analysis included sections taken at the level of the mid striatum, 26 mm anterior to stereotaxic zero according to the fetal sheep stereological atlas^54^, with two adjacent sections selected from each animal (levels were matched between animals) for each analysis.

Sections were dewaxed for 2 × 15 min in 100% xylene, and rehydrated by immersion in decreasing concentrations of ethanol for 5 min each (100%, 90%, and 75%). Following 3 × 5 min washes in 0.1 M phosphate buffered saline (PBS), antigen retrieval was performed in 10 mM citrate buffer at >120 °C using the pressure cooker method (Antigen 200 Retriever; Electron Microscopy Sciences, Emgrid, Australia), and sections were again washed for 3 × 5 min in PBS. Sections were then incubated in 1% hydrogen peroxide in methanol to block endogenous peroxidase activity. Sections were washed for 3 × 5 min in PBS, then blocked in 5% normal goat serum (NGS)/PBS for 1 h at room temperature, followed by incubation with rabbit anti-glutamate decarboxylase (GAD)65/67 (1:200; #ab49832; Abcam, Cambridge, MA, USA) overnight at 4 °C. After 3 × 5 min washes in PBS, sections were incubated in biotin-conjugated goat anti-rabbit IgG secondary antibody (1∶200) in 3% NGS/PBS for 3 h at room temperature, washed for 3 × 5 min in PBS, and then incubated in ExtrAvidin®-Peroxidase (1∶200; #E2886; Sigma-Aldrich, St. Louis, MO, USA) in PBS for 2 h at room temperature. Sections were washed for 3 × 5 min in PBS, and then antibodies visualised by incubation in 3,3′-diaminobenzidine tetrahydrochloride hydrate (DAB; #D4293; Sigma-Aldrich). The reaction was stopped by washing in distilled water, and sections were dehydrated in increasing concentrations of ethanol (75%, 90%, 100%, 5 min each), followed by 2 × 10 min incubations in xylene, and then coverslipped with DPX mounting media (Sigma-Aldrich).

#### Fluorescent labelling

For multiple-labelling experiments, mid-striatal brain sections were selected, and sections were dewaxed, rehydrated, and antigen retrieved as described above. For detection of PNN expression (biotinylated WFA; 1:400; #L1516; Sigma-Aldrich), sections were incubated for 15 min in 0.1% avidin/PBS, and then 15 min in 0.1% biotin/PBS, for blocking endogenous biotin. For all other primary antibodies, including rabbit anti-GAD65/67 (1:200; #ab49832; Abcam), rabbit anti-parvalbumin (PV; 1:50; #ab11427; Abcam), mouse anti-NeuN (1:20; #MAB377; Merck Millipore, Billerica, MA, USA), and chicken anti-myelin basic protein (MBP; 1:500; #MBP; Aves Labs, Tilgard, OR, USA), sections were incubated in 0.1 M glycine for 20 min to reduce tissue autofluorescence, washed for 3 × 5 min in PBS, and blocked in 5% NGS. Sections were then incubated with WFA and primary antibodies in PBS/3% NGS for 3 nights at 4 °C, washed for 3 × 5 min in PBS, followed by appropriate secondary antibodies (Thermo Fisher Scientific, Waltham, MA, USA), including goat anti-rabbit Alexa Fluor 488 or 594 (1:200), goat anti-mouse Alexa Fluor 647 IgG_1_ (1:100; #A-21240; used to detect NeuN primary antibody), streptavidin-conjugated Alexa Fluor 594 (1:200) or 680 (1:200), goat anti-chicken fluorescein conjugate (#F-1005; Aves Labs), and Hoechst 33258 (1:10,000; Thermo Fisher Scientific), for 2.5 h at room temperature. Slides were washed again for 3 × 5 min in PBS, and coverslipped using Vectashield mounting medium (Vector Laboratories, Burlingame, CA, USA).

For calmodulin-dependent protein kinase IIα (CaMKIIα) immunolabelling, paraffin-fixed sections were processed as above until the NGS blocking step, except the glycine step was omitted. Sections were then incubated in rabbit anti-CaMKIIα (1:200; #C6974; Sigma-Aldrich) for 3 nights at 4 °C, and then washed for 3 × 5 min in PBS. To amplify the CaMKII signal, biotin-conjugated goat anti-rabbit antibody (1:200; Vector Laboratories) was added for 3 h at room temperature, followed by washing and incubation in streptavidin-conjugated Alexa Fluor 488 (1:200) for 2.5 h at room temperature. Sections were washed and a second avidin-biotin block was performed, and sections were then incubated in WFA (1:400) overnight at 4 °C. Finally, sections were washed, streptavidin-conjugated Alexa Fluor 594 and Hoechst 33258 were added for 2.5 h, followed by washing and coverslipping as above.

For immunolabelling with cortical layer markers, brain sections were immersion-fixed in 4% paraformaldehyde, cut at 50 µm using a freezing microtome, and stored in cryoprotectant at −20 °C. Free-floating sections were washed for 3 × 5 min in PBS, incubated in 10 mM citrate buffer at 85 °C for 5 min for antigen retrieval, cooled for 20 min at room temperature, and then washed for 3 × 5 min in PBS. Sections were blocked for 1 h in 5% normal donkey serum (NDS; Thermo Fisher Scientific)/PBS at room temperature, and then double-labelled with rat anti-CTIP2 (1:500; #ab18465; Abcam) and biotinylated WFA (1:400) in 3% NDS/PBS for 3 nights at 4 °C. Sections were washed for 3 × 5 min, and incubated in donkey anti-rat Alexa Fluor 594 (1:500) and streptavidin-conjugated Alexa Fluor 680 (1:250) secondary antibodies, with Hoechst 33258 (1:10,000), in 3% NDS/PBS for 2.5 h at room temperature. After 3 × 5 min PBS washes, sections were mounted and coverslipped.

### Imaging and analysis

#### Quantification of WFA, GAD, and PV expression in fluorescent sections

**Figure 1:**
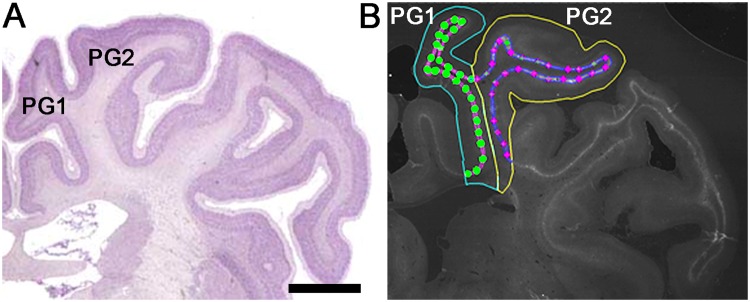
Sampling regions in the parasagittal cortex of the near-term fetal sheep brain. (**A**) Sheep brain atlas showing the first (PG1) and second (PG2) parasagittal gyri at the mid-striatal level. (**B**) Representative tracing and sampling sites of the *Wisteria floribunda* agglutinin^+^ (WFA^+^) layer for PG1 and PG2. The WFA^+^ layer is traced in pink (PG1) or blue (PG2), while the sampling sites are marked with circles (PG1) or diamonds (PG2). (**A**) was adapted with permission from http://www.brains.rad.msu.edu, supported by the US National Science Foundation and the National Institutes of Health. Scale bar: 5 mm.

Using imaging software (Stereo Investigator; MBF Bioscience, Williston, VT, USA) driving a motorised stage (MAC 6000, MBF Bioscience) connected to a microscope (Zeiss AxioImager M2; Carl Zeiss Microscopy, LLC, Thornwood, NY, USA), the first and second parasagittal gyri (PG1 and PG2, respectively) of the right hemisphere (the left hemisphere was used in cases where there was damage to the right hemisphere) of each brain section were traced (2.5× objective), using the common sulcus as a guide for dividing the two gyri (Fig. 1). The dense WFA immunoreactive layer was then traced (5× objective) for each gyrus using WFA, NeuN, and Hoechst labelling as guides. The inner boundary of this layer was adjacent to the border of the white matter, defined using Hoechst and NeuN. The numbers of WFA^+^, GAD^+^, PV^+^, and CaMKII/WFA^+^ neurons in this layer were counted (40× objective; counting frame size: 100 µm × 100 µm; ~25 sites per gyrus) for each gyrus, beginning at the intersection between PG1 and PG2. Positive cells were selected based on the following criteria: (1) a nucleus size within two standard deviations of the mean control nuclear size (based on data from ~20 control neurons per animal), and (2) a staining pattern and morphology similar to control neurons. Cell somata touching either of the two inclusion lines of the counting frame were included, while somata touching either of the two exclusion lines were not counted. Counting frames with greater than one-third falling outside of the traced boundary were excluded, as were regions with evidence of marked tissue loss. The densities of WFA^+^, GAD^+^, and PV^+^ cells (per mm^2^) were calculated for each gyrus, and two slides per animal were averaged to obtain final data.

#### Quantification of GAD+ cells in the parasagittal cortex in DAB labelled sections

The cortices of PG1 and PG2 were traced and counted as described above. The numbers of GAD^+^ neurons in all layers of the parasagittal cortex were counted using the fractionator probe (grid size: 500 µm × 500 µm; counting frame size: 150 µm × 150 µm; ~25 sites per gyrus) for each gyrus. The density of GAD^+^ cells (per mm^2^) was calculated for each gyrus, and two slides per animal were averaged to obtain final data.

### Data analysis

Off-line physiological data analysis was performed using LabVIEW based customised programs. Seizures were identified visually and defined as sudden repetitive and evolving waveforms in the EEG signal lasting more than 10 s and of an amplitude greater than 20 μV^55^. All biochemical, neurophysiological, cardiovascular, and fetal growth data from this cohort of animals were previously reported^46^. For neuronal count data, a two-way ANOVA was performed to test for overall differences in cell densities in PG1 and PG2 of control and HI animals. Where an overall effect was found, a Fisher’s least squared derivative test was performed to assess for differences between the groups. Linear regression analysis was used to compare the relationship between loss of GAD^+^ neurons or PNNs in cortical layer 6, and seizure burden following HI. All statistical analyses were performed with statistical software (GraphPad Prism; GraphPad software, La Jolla, CA, USA). Statistical significance was accepted at *p* < 0.05. Data are presented as mean ± standard deviation. Histology and neuronal counts were performed by an assessor (T.M.F.) who was blinded to the treatment groups.

## Results

### PNN and interneuron localisation in uninjured sheep brain

**Figure 2:**
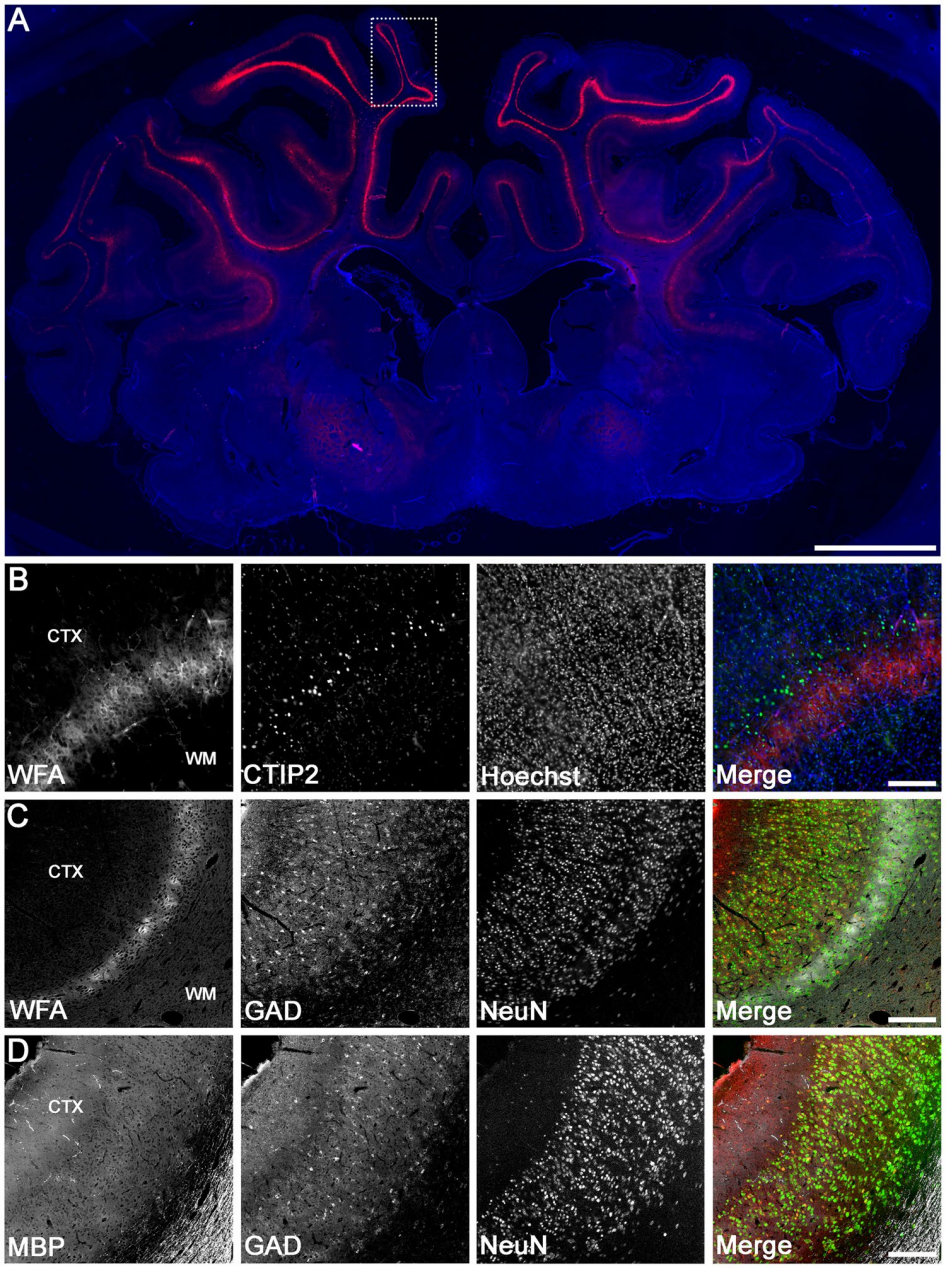
Localisation of WFA staining and interneurons in the uninjured near-term sheep cortex. (**A**) WFA reactivity in the whole brain (red) co-labelled with Hoechst 33258 (blue). Representative high magnification examples of co-labelling of WFA (red) and CTIP2 (green) (**B**), WFA (white), glutamic acid decarboxylase (GAD, red), and NeuN (green) (**C**), and myelin basic protein (MBP, white), GAD (red), and NeuN (green) (**D**) taken from the boxed area in PG1 (**A**). CTX, cortex. Scale bar: (**A**), 5 mm; (**B**–**E**), 200 µm.

**Figure 3:**
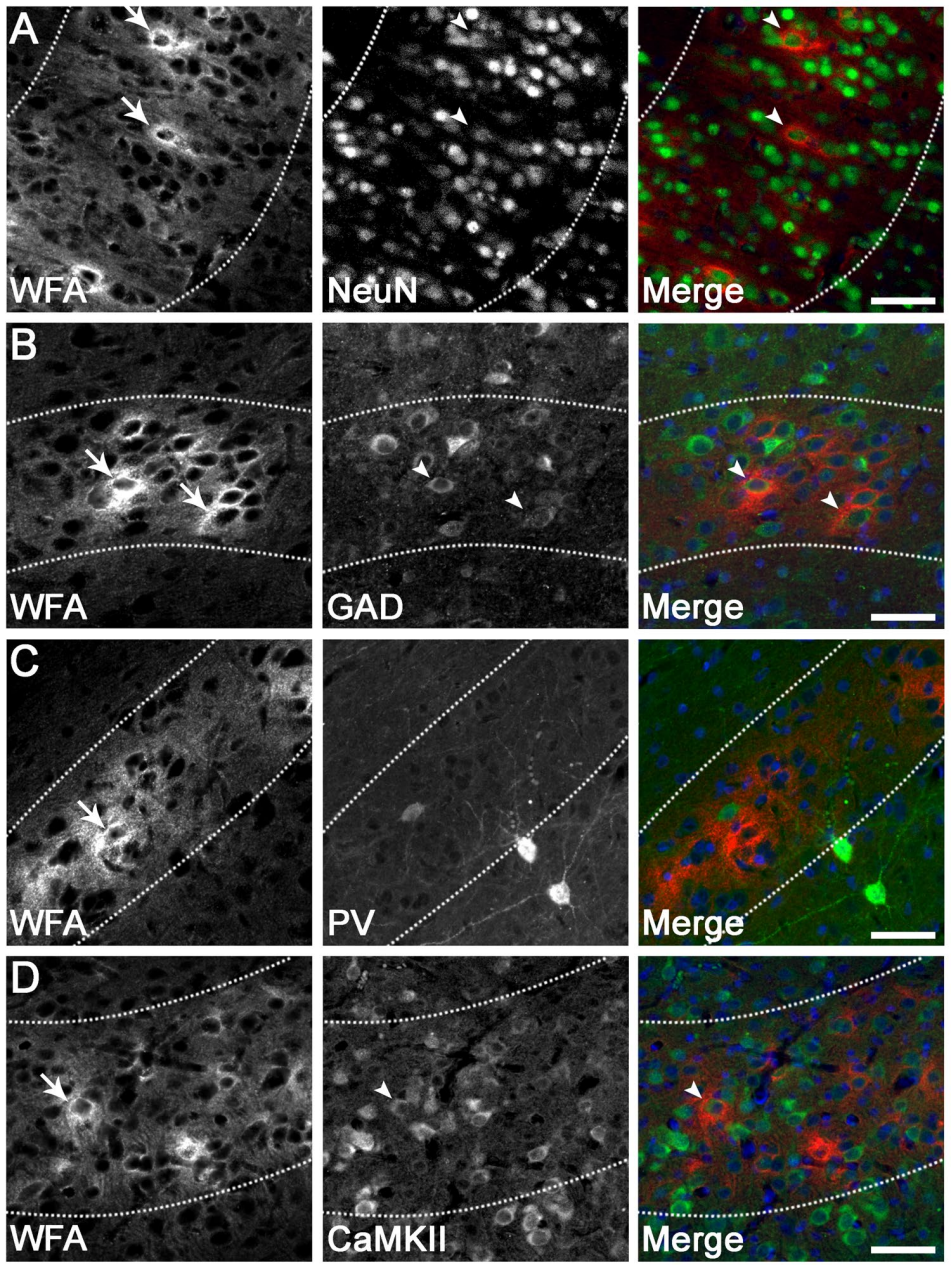
PNN expression on various neuronal subtypes in the parasagittal cortex. Dense pericellular WFA reactivity (arrows) was observed around subsets of NeuN^+^ neurons (**A**; arrowheads), GAD^+^ interneurons (**B**; arrowheads), and CaMKIIα^+^ excitatory neurons (**D**; arrowheads), in a pattern resembling immature PNNs. By contrast, very few PNNs (arrows) were localised to parvalbumin (PV)^+^ interneurons (**C**). Note that the diffuse extracellular component of WFA staining is observed within layer 6 (dotted lines). Scale bar: 50 µm.

We first determined the pattern of WFA labelling in control near-term fetal sheep brains. Intense WFA staining was observed in the cerebral cortex (Fig. 2A), particularly in the parasagittal cortex (including both PG1 and PG2), and largely confined to the infragranular layers. Within these regions, WFA staining included a pattern of dense pericellular labelling around a subpopulation of somata (e.g., Fig. 3A,B [arrowheads]), as well as a more diffuse extracellular component (e.g., Fig. 3A,B [layer 6 region defined within dotted lines]). Co-labelling with the cortical layer marker CTIP2, which is most strongly expressed in cortical layer 5^56,57^, revealed predominant WFA labelling in cortical layer 6 (i.e., directly below the layer of highest CTIP2 expression). Immunohistochemistry for NeuN (post-mitotic neuron marker) and MBP (myelin marker) confirmed that the WFA layer was adjacent to the white matter (Fig. 2C,D). We also assessed GAD expression in the parasagittal cortex, and found that GAD^+^ neurons were distributed throughout all cortical layers, including the WFA-rich layer 6 (Fig. 2C,D).

As expected, all WFA^+^ cells co-expressed the neuronal marker NeuN (Fig. 3A), and exhibited a pattern resembling immature PNNs, with dense immunoreactivity surrounding the soma and occasionally proximal processes (by contrast, mature PNNs surround proximal processes more frequently, and often extend further along processes)^28,30,58^. Double labelling revealed that 20.1 ± 8.8% (PG1) and 15.2 ± 4.5% (PG2) of total PNNs were expressed on GAD^+^ interneurons in layer 6 (e.g., Fig. 3B), while 6.6 ± 7.2% (PG1) and 4.3 ± 3.4 (PG2) of total PNNs were expressed on PV^+^ interneurons in layer 6 (e.g., Fig. 3C). Note that the levels of neuronal GAD and PV expression varied from weak to strong. Finally, 7.2 ± 6.2% (PG1) and 20.3 ± 15.5% (PG2) of total PNNs were expressed on CaMKIIα^+^ excitatory neurons in layer 6 (e.g., Fig. 3D).

### Loss of total interneurons in the parasagittal cortex following term HI

**Figure 4:**
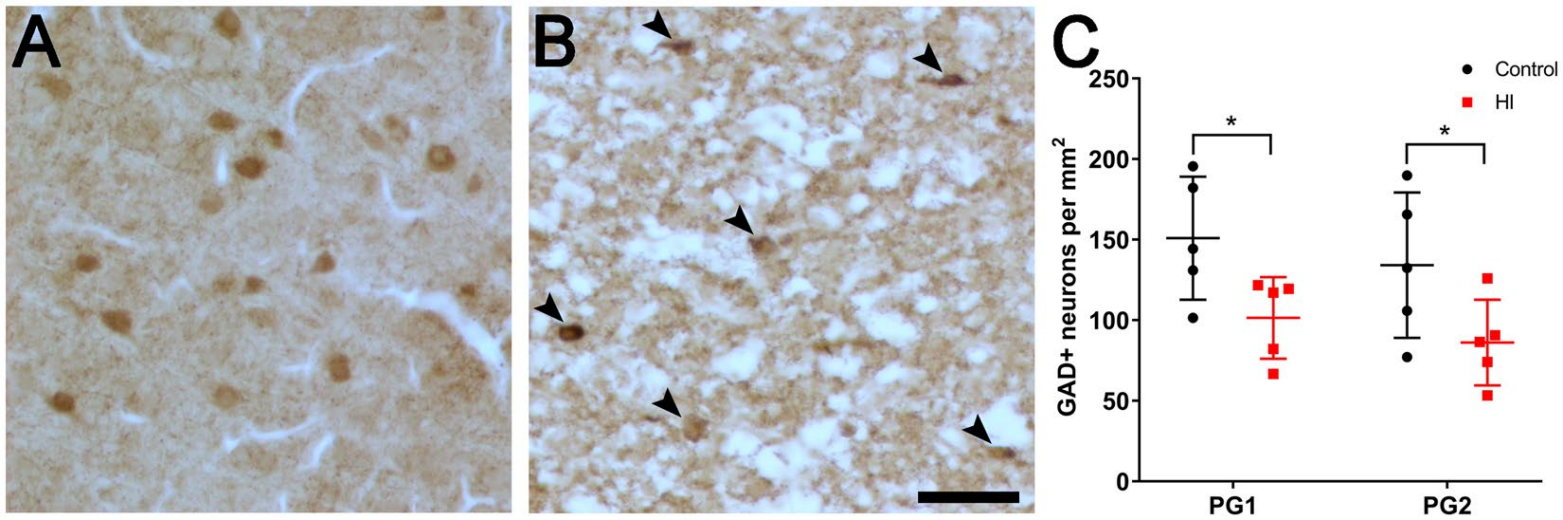
GAD expression in all layers of the parasagittal cortex in PG1 and PG2 of control (n = 5) and hypoxia-ischaemia (HI, n = 5) animals at 7 d recovery. Representative images of (**A**) control and (**B**) HI tissue in PG1 showing a reduction in GAD^+^ neurons (brown cells) in the HI cortex. GAD^+^ neurons in (**B**) are indicated with arrowheads. (**C**) Density of GAD^+^ neurons in PG1 and PG2 of control and HI animals. *p < 0.05. Scale bar: 50 µm. Data are mean ± standard deviation.

To quantify the effect of HI on cortical interneuron survival, we assessed the density of GAD^+^ neurons in all layers of the parasagittal cortex in PG1 and PG2 (see tracing boundaries in Fig. 1B) at 7 d recovery after HI in near-term fetal sheep. There was a significant reduction in the density of GAD^+^ neurons in both PG1 (Control: 151 ± 38 vs. HI: 101 ± 25 cells/mm^2^; *p* = 0.0391) and PG2 (Control: 134 ± 45 vs. HI: 86 ± 27, respectively; *p* = 0.0443) in HI animals (Fig. 4). There were no significant differences in GAD^+^ cell densities between PG1 and PG2 for the control or the HI groups.

### Loss of PNNs in layer 6 of the parasagittal cortex following term HI

**Figure 5:**
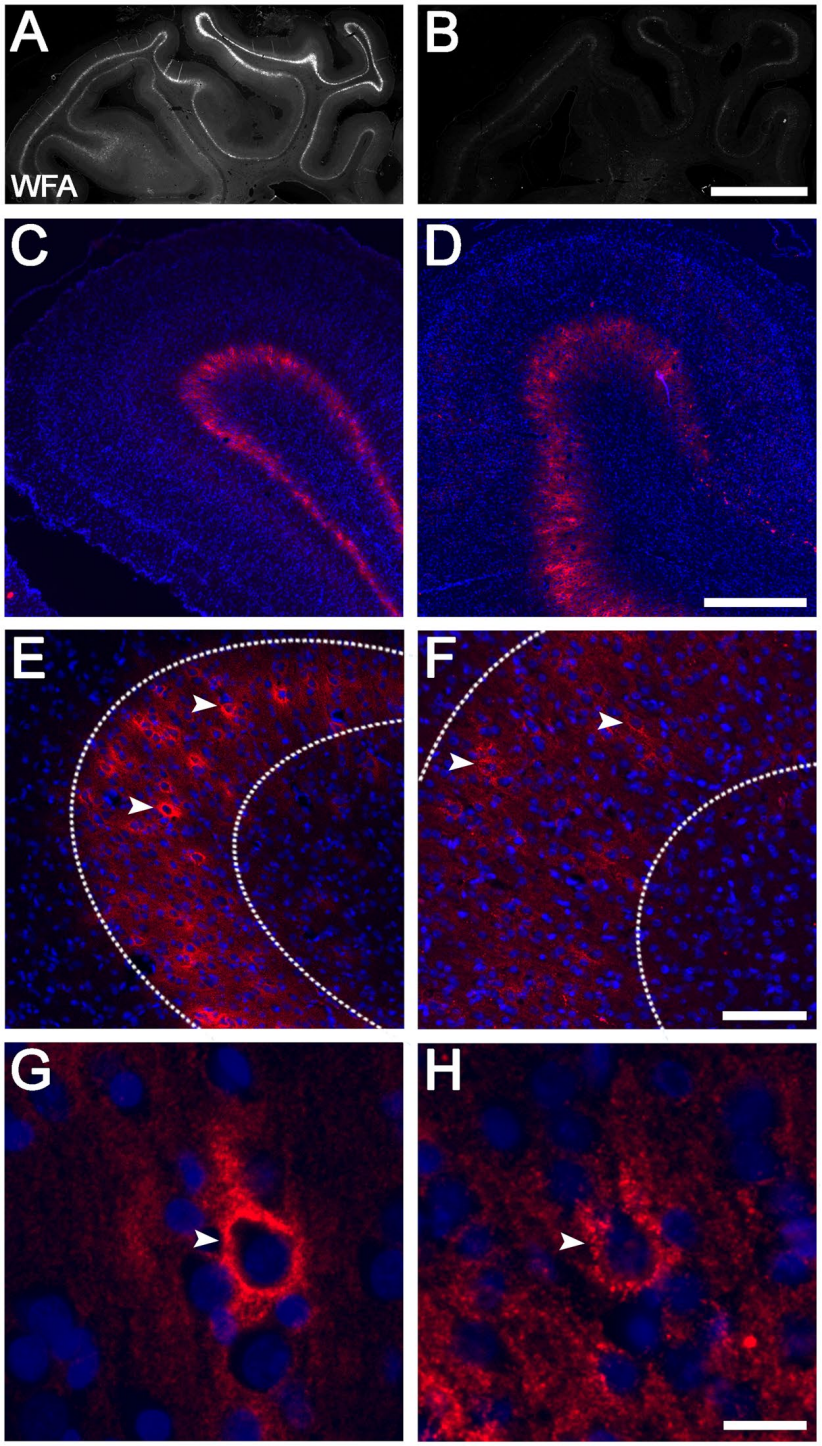
Representative examples of PNNs in the parasagittal cortex of control and HI animals at 7 d recovery. Control (left column), HI (right column). WFA (**A**,**B**: grey, **C**–**H**: red), Hoechst 33258 (blue). Note that because of the marked reduction in WFA staining in HI animals, the image intensity of injured tissues (**D**,**F**,**H**) was manually adjusted (four-fold increase relative to control images) to allow visualisation of staining patterns. Representative examples of actual WFA staining levels between control and HI animals are shown in panels A and B. Images (**C–H**) were taken from PG1. Images (**E–H**) were taken from layer 6. Arrowheads indicate PNNs. The diffuse extracellular WFA staining is observed within layer 6 indicated by dotted lines. Scale bars: (**A**,**B**) 5 mm, (**C, D**) 500 µm, (**E, F**) 100 µm, (**G, H**) 20 µm.

Next, we assessed PNN expression in layer 6 of the parasagittal cortex following HI. There was a marked overall decrease in WFA staining intensity in layer 6 of PG1 and PG2 after HI (Fig. 5B) compared with control animals (Fig. 5A), with complete loss in some regions (e.g., Fig. 5C,D). Areas with reduced but visible WFA reactivity were predominantly restricted to the superior surface of PG1 and PG2. Interestingly, the remaining PNNs in layer 6 of the injured PG1 and PG2 cortices were often less sharply defined (i.e., more diffuse), with reduced pericellular staining and lower WFA intensity compared with controls (Fig. 5E–H).

**Figure 6:**
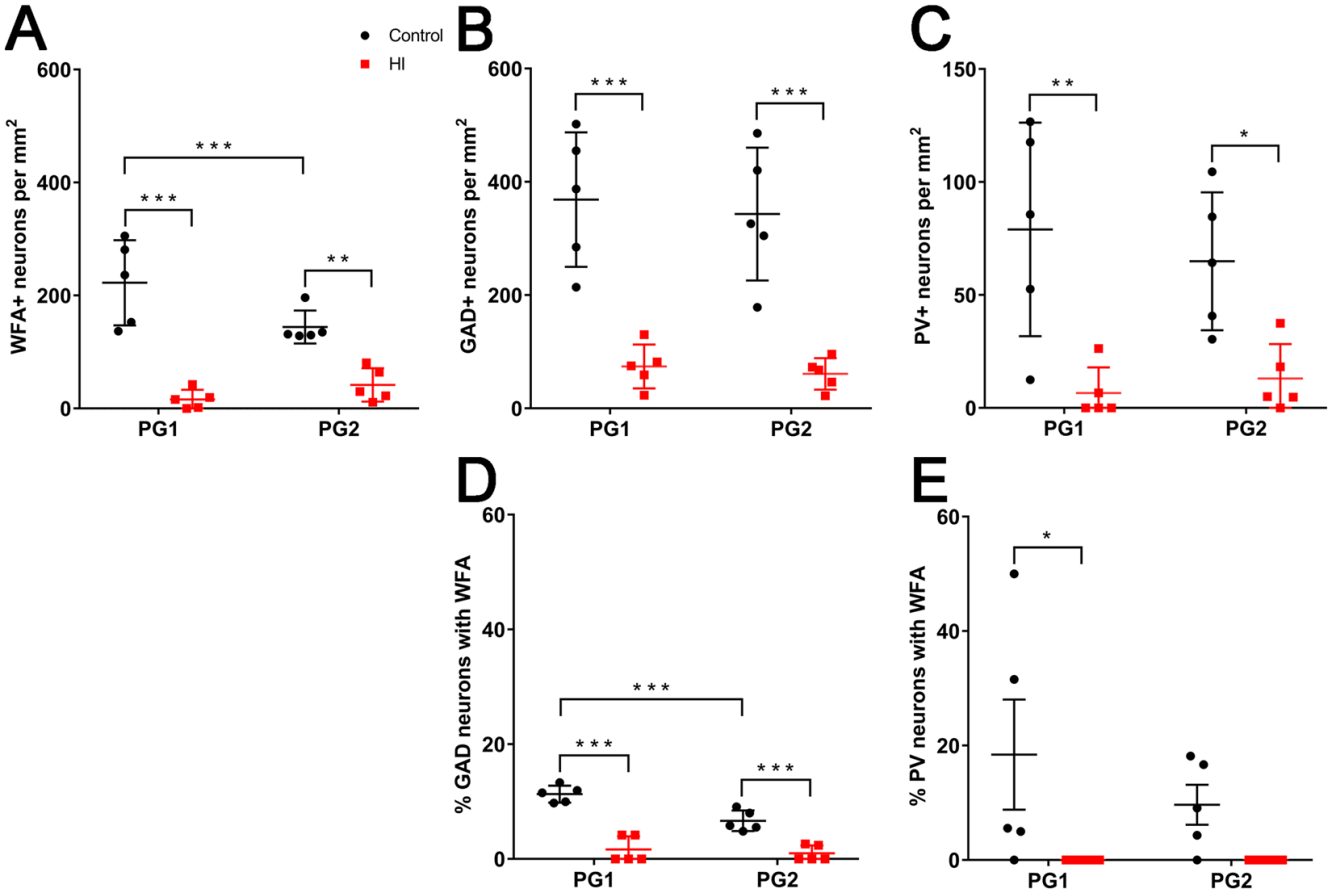
Density of PNNs, and GAD^+^, and PV^+^ interneurons, in the parasagittal cortex of control and HI animals at 7 d recovery. Density of (**A**) WFA^+^, (**B**) GAD^+^, and (**C**) PV^+^ neurons in cortical layer 6. (**D**) Proportion of total GAD^+^ neurons that were WFA^+^. (**E**) Proportion of total PV^+^ neurons that were WFA^+^. n = 5 animals/group. *p < 0.05, **p < 0.01, ***p < 0.001. Data are mean ± standard deviation.

We then assessed the effect of HI on the number of PNNs in cortical layer 6. In HI animals there was a significant reduction in the density of total WFA^+^ neurons compared with controls in both PG1 (Control: 223 ± 75 vs. HI: 16 ± 17 cells/mm^2^, *p* < 0.0001) and PG2 (Control: 144 ± 29 vs. HI: 42 ± 30 cells/mm^2^, *p* = 0.0019) (Fig. 6A). Next, we examined whether this decrease in WFA^+^ cell density was because of interneuron death or loss of PNNs on surviving interneurons. HI was associated with a significant reduction in the density of GAD^+^ neurons (all GABAergic interneurons) and PV^+^ neurons within layer 6 in PG1 (GAD^+^: 369 ± 119 vs. 74 ± 39 cells/mm^2^, *p* < 0.0001; PV^+^: 79 ± 47 vs. 7 ± 11 cells/mm^2^, *p* = 0.0014) and PG2 (GAD^+^: 343 ± 117 vs. 61 ± 28 cells/mm^2^, *p* < 0.0001; PV^+^: 65 ± 31 vs. 13 ± 15 cells/mm^2^, *p* = 0.0139) compared with controls (Fig. 6B,E). Double-labelling for GAD/WFA^+^ and PV/WFA^+^ neurons in control animals showed that approximately 11% (PG1) and 7% (PG2) of the total GAD^+^ cell population expressed PNNs (Fig. 6C), and approximately 18% (PG1) and 10% (PG2) of the total PV population expressed PNNs (Fig. 6C). By contrast, HI was associated with reduced WFA colocalisation with GAD^+^ (to 1.7% for PG1 and 1.0% for PG2, *p* ≤ 0.0001 for both) and PV^+^ (to 0% for both PG1 [*p* = 0.0217] and PG2, [*p* = 0.2011]) cells, indicating that PNNs were also lost on surviving interneurons.

### Loss of cortical interneurons and PNNs is not significantly associated with increased electrographic seizure burden

**Figure 7:**
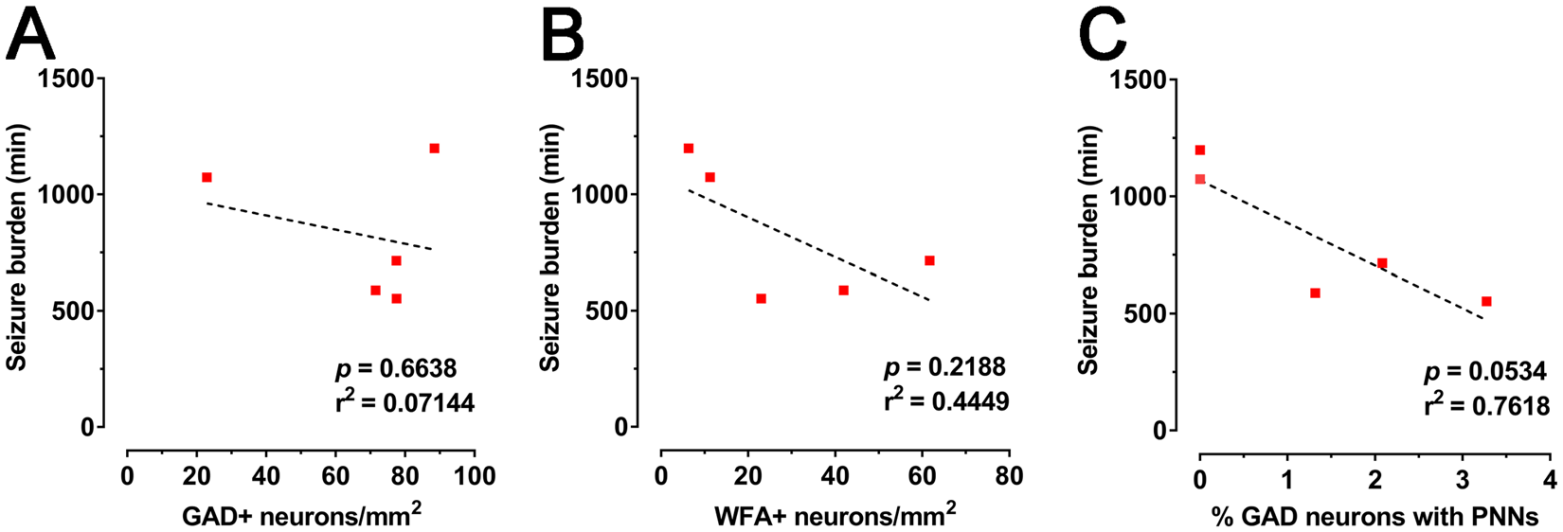
Relationship between loss of GAD^+^ neurons or PNNs in layer 6 of the parasagittal cortex with seizure activity in HI animals at 7 d recovery. Linear correlations of (**A**) GAD^+^ cell density, (**B**) WFA^+^ density, and (**C**) the proportion of GAD^+^ neurons with PNNs with seizure burden are shown.

Finally, we examined the relationship between the density of GAD^+^ neurons or PNNs in cortical layer 6, and seizure burden in the HI animals. There was no significant linear correlation of GAD^+^ cell density (*p* = 0.6638; Fig. 7A), WFA^+^ cell density (*p* = 0.2188; Fig. 7B), or the proportion of GAD^+^ neurons that were WFA^+^ (*p* = 0.0534; Fig. 7C) with seizure burden.

## Discussion

Disrupted GABAergic signalling in the cerebral cortex is thought to contribute to the neurophysiological and cognitive impairments observed in numerous neurodevelopmental disorders, and there is limited preclinical and human evidence of damage to cortical GABAergic interneurons after perinatal brain injury^24–27^. Herein, we show that HI in the near-term fetal sheep (equivalent to the term human with respect to brain development)^47,48^ results in marked loss of GABAergic interneurons throughout the parasagittal cortex. We also provide new evidence for formation of PNNs on cortical interneurons during the last third of gestation in the fetal sheep, localised mainly to cortical layer 6. This population of cortical interneurons was particularly vulnerable to injury following HI, with widespread interneuron and PNN loss in layer 6, along with disruption of PNNs on surviving neurons. Overall, these data suggest that perinatal HI may cause deficits in the cortical GABAergic system that involve, at least in part, disruption of PNNs that are important for cortical inhibitory network function and regulation of CNS plasticity.

Injury to the parasagittal cortex is a common pattern of brain damage observed following HI in term infants. This pattern of injury is reproduced in near-term animal models of cerebral HI, including the 0.85 gestation fetal sheep^3,50^. Our findings demonstrate that HI near term causes marked loss of GABAergic interneurons (GAD^+^: by ~34%) throughout the parasagittal cortex at 1 week of recovery. While these changes may relate to downregulation of interneuron markers on surviving neurons rather than cell death, this is unlikely as the degree of interneuron loss in the parasagittal cortex following HI in the present study was similar to the total cortical neuronal loss we previously reported for this cohort^46^. Interneuron loss was even more pronounced in cortical layer 6 (GAD^+^: by ~88%; PV^+^: by ~86%), suggesting a particular vulnerability of this cell layer. Previous reports showed that unilateral HI caused acute death of GAD^+^ interneurons in the cerebral cortex in postnatal day (PND)9 mice^26^, while asphyxia in newborn rats (PND0) resulted in long-term (PND56) loss of cortical calbindin^+^ interneurons (~20%) in the frontal cortex^27^. Further, maternal hypoxia at embryonic day 17 in mice caused an acute, but transient, reduction of GAD protein expression in the cerebral cortex, and a reduced density of cortical calbindin^+^ interneurons in both superficial and deep layers by PND14^25^. Despite no equivalent reports in term humans, post-mortem studies in preterm born infants with white matter lesions (25–32 weeks gestation) show a reduced density of cortical calretinin^+^ interneurons and reduced numbers of cortical cells with GABA receptor expression^24^. Overall, these studies in combination with our observations indicate that injury to cortical interneurons may form an important component of the neuropathology observed following perinatal HI.

In the present study, HI resulted in loss of PNNs in layer 6 (by ~85%), which, at least in part, reflects the loss of cortical interneurons. Nevertheless, there was also a marked reduction in the expression of PNNs on surviving GAD^+^ (by ~85%) and PV^+^ (by 100%) interneurons, while remaining PNNs showed overall weaker and less defined staining, indicative of reduced PNN integrity. Similarly, expression of brevican, a major PNN component, was reported to be reduced in the cortex after HI in neonatal rats^59^. Loss of cortical PNNs was also shown after stroke in the adult rat^44^, which was attributed to degradation of PNN components. In support, expression of matrix metalloproteinase 9 (MMP9), which is particularly important for PNN degradation, was increased after HI in the neonatal mouse cortex^60^, and after focal ischaemia in the adult rat cortex^61–63^. ADAMTS (a disintegrin and metalloproteinase with thrombospondin motifs) and hyaluronidase (Hyal) enzymes also degrade PNNs, and are upregulated in the brain of adult humans and rats after focal ischaemia^64–66^. In these studies, upregulation of MMP, Hyal, and ADAMTSs occurred rapidly (2–24 h) after injury, and often persisted for days. Thus, the reduction in cortical PNNs with HI in the present study may reflect an increase in enzymatic PNN degradation. Further studies examining the exact timing of PNN production during fetal life and expression of PNN-degrading enzymes after HI will aid in determining the mechanisms underlying these changes.

Numerous studies have reported key roles of GABAergic neurons in normal cortical circuit development^17,21^, and of PNNs in GABAergic network function^32–34^. Deficits in cortical GABAergic signalling cause an imbalance in neural excitatory-inhibitory activity^67^, and can produce phenotypes similar to those observed in children following perinatal brain injury, including altered cortical plasticity and excitability, epilepsy, cognitive delay, and behavioural dysfunction^68–74^. Even though we did not observe a relationship between loss of interneurons or PNNs and seizure burden following HI in the present study, others showed that degradation of PNNs in hippocampal cultures i*n vitro* caused increased interneuron excitability and seizure-like activity^32,75^. Further, loss of PNNs may increase interneuron vulnerability to oxidative stress and death^76–78^. Thus, we suggest that changes in cortical interneuron and PNN expression may still contribute to the cortical hyperexcitability, seizures, and delayed neuronal loss previously reported in this model^46,50^. Greater animal numbers are required to confirm the relationship between loss of interneurons or PNNs and seizure burden. Loss of GABAergic interneurons and PNN disruption around term age is also likely to have longer-term implications for cortical function. For example, GABAergic circuit establishment is the trigger for the beginning of critical periods of cortical development (periods during which synaptic circuitry is most easily modified by experience), while PNNs are required for restriction of plasticity and critical period closure^79,80^. Further studies are required to examine the longer-term effects of interneuron and PNN injury following perinatal HI.

PNNs are known to exhibit developmental and regional changes in their cortical expression patterns. Although there are no previous reports of fetal PNN expression, in humans, immature PNNs were first observed in the medial prefrontal cortex at 2 months of postnatal age, reaching mature levels by 8 years^81^. In rats, immature PNNs first develop in layer 6 of the parietal cortex at PND7, an age of brain maturation equivalent to the late gestation human^82,83^, followed by more widespread PNN expression in cortical layers 2–6 by PND14, and then adult-like patterns by PND35^84^. A similar timing of PNN formation was shown in the mouse visual cortex, but PNNs were highest in layers 4 and 5, and lower in layers 2/3 and 6 at all ages (PND10–PND70)^85^. In adult sheep, PNNs are predominantly located in layers 3 and 5, with less in layers 4 and 6^86^, while in adult humans and monkeys, PNNs are expressed in cortical layers 3 and 4, with less in layers 2, 5, and 6^39,87^. The initial appearance of PNNs in cortical layer 6 in the present study likely reflects the earlier formation of this layer compared with other cortical lamina, as during cortical development, the deeper layers (5 and 6) form first, followed by superficial layers (2–4)^57^. In sheep, neurogenesis and formation of cortical layers 5 and 6 occur from approximately 30 d gestation, and are largely complete by 60 d^88^.

We found that only a low proportion of PNNs in layer 6 were localised to inhibitory GABAergic neurons identified using GAD (~18%) or PV (~6%), which contrasts with adult human and animal studies showing a majority of cortical PNNs (up to 87%) on interneurons^89,90^. These adult studies also imply that the maximum proportion of PNNs localised to excitatory neurons is ~13%, which is similar to that observed on excitatory neurons identified using CaMKIIα (~14%) in the present study. Thus, in the near-term fetal sheep brain, there remain numerous PNNs expressed on neurons that do not express common inhibitory markers. In support, initial appearance of PNNs and PV^+^ interneurons was reported to occur earlier than their colocalisation during postnatal development in the human prefrontal cortex^42^. These findings may relate to the ongoing maturation of cortical inhibitory circuity during fetal and postnatal periods. Cortical interneuron fate, including the interneuron subtype, is specified during fetal life, prior to cell migration from the ganglionic eminences, while the differentiation of these neurons (including expression of various interneuron markers) then occurs once the cells reach their cortical destination^91,92^. In humans, the migration of GABAergic interneurons into the cerebral cortex continues into early postnatal life (~6 months of age)^22^. Further, in many species, including rodents, cats, and humans, the expression of GABA, GAD, and PV in the cerebral cortex does not reach adult levels until around adolescence (e.g., postnatal day 15–21 in the rodent, 5 weeks postnatally in the kitten, and 12–20 years in the human)^93–95^; human cortical GAD expression during late gestation is only <10% of adult levels, and then progressively increases postnatally and into adulthood^22^. Thus, the total proportion of PNNs on interneurons during early development may be higher than that observed by GAD and PV labelling; i.e., there may be neuronal populations with mRNA expression of GABAergic interneuron markers that do not yet express the proteins.

In summary, our data suggest that perinatal HI causes deficits in the cortical GABAergic system involving loss of interneurons and disruption of PNNs. Given the importance of GABAergic networks in CNS maturation and function, these deficits may contribute to the wide range of adverse neurological outcomes associated with cerebral HI.
